# The Antidepressant-Like Effect of Lactate in an Animal Model of Menopausal Depression

**DOI:** 10.3390/biomedicines6040108

**Published:** 2018-11-21

**Authors:** Noof Abdullah Shaif, Daehyuk Jang, Donghyun Cho, Sunmi Kim, Dae Bang Seo, Insop Shim

**Affiliations:** 1 Department of Science in Korean Medicine, College of Korean Medicine, Kyung Hee University, Seoul 02447, Korea; n.love9999@yahoo.com (N.A.S.); nisclist@nate.com (D.J.); 2 Vital Beautie Research Division, AMOREPACIFIC R&D Unit, Gyeonggi-do 17074, Korea; cdh0529@amorepacific.com (D.C.); apsun20@amorepacific.com (S.K.); sdbang@amorepacific.com (D.B.S.); 3 Department of Physiology, College of Medicine, Kyung Hee University, Seoul 02447, Korea

**Keywords:** lactate, ovariectomy, depression, tyrosine hydroxylase, tryptophan hydroxylase, protein kinase C, neurotransmitters, serotonin and dopamine

## Abstract

Background: This study aimed to investigate the antidepressant-like effect of lactate and elucidate its mechanisms in ovariectomized rats with repeated stress. Methods: Two experiments were conducted on female rats in which all groups, except normal, were ovariectomized and underwent immobilization for 14 days. Lactate was administered orally (100, 250, and 500 mg/kg) for 14 consecutive days, and the rats’ cutaneous body temperature was measured during the same period. Depression-like behavior in rats was assessed by the tail suspension test (TST) and forced swimming test (FST). Furthermore, enzyme-linked immunosorbent assay (ELISA) and immunohistochemistry were conducted to evaluate the changes that occurred in the neurotransmitter levels and activity. Results: The lactate 100 and 250 groups had reduced time spent immobile in TST and FST and decreased peripheral body temperature. In ELISA tests, the lactate 250 group expressed elevated levels of serotonin and dopamine in many brain areas. Tyrosine hydroxylase (TH), tryptophan hydroxylase (TPH), and protein kinase C (PKC) immunoreactive cells showed increased density and cell counts in lactate administered groups. Conclusion: Results indicated that lactate has an antidepressant effect that is achieved by activation of PKC and upregulation of TH and TPH expression, which eventually leads to enhanced serotonin and dopamine levels in the menopausal rat’s brain.

## 1. Introduction

The World Health Organization recently published a report indicating that major depressive disorders (MDD) are affecting 322 million people all around the world, and the prevalence among women is higher than in men by one and a half to three times. Particularly, the difference in the incidence was noticed to be higher around the time of menopause indicating a hormonal impact that increases the risk of depression [1].

Menopause is defined as absolute cessation of menstrual cycles in women followed by one year of amenorrhea. Menopause could be as a result of lost ovarian follicles as a natural aging process, from undergoing surgeries like ovariectomy or hysterectomy, or due to premature idiopathic ovarian failure [2].

For years, numerous researchers have tried to explain the pathophysiology of menopausal depression by estrogen withdrawal theory. This theory suggests that diminished estrogen levels affect brain neurotransmitters, such as dopamine, norepinephrine, and serotonin [2,3].

The majority of women with menopause show symptoms—other than depression—like hot flushes (HF) [3]. It is reported that diminished estrogen hormones, along with abnormal norepinephrine (NE) levels, cause thermoregulation and vascular dysfunction which leads to the occurrence of HF [4,5]. Menopausal women with HF experience sleep disturbance which, in turn, leads to depression and mood changes [6].

To date, it is well-known that normal levels of estrogen hormone (17β-estradiol) regulates central monoaminergic neurotransmitters like serotonin and dopamine [7] via enhancing the expression of tryptophan hydroxylase (TPH) and tyrosine hydroxylase (TH) in the brain [8,9]. Additionally, it is worth mentioning that a normal level of estrogen enhances protein kinase C (PKC) activity and expression [10]. In the brain, PKC has a critical role in regulating the pre- and post-synaptic membrane, neurotransmitter release, neuronal signaling and plasticity, and in protecting neurons from cell death [10,11]. Many studies have reported that an absence of estrogen hormone and/or stress induced a downregulation of PKC expression, especially in the hippocampal area, is believed to be involved in the pathology of depressive disorders [12,13].

Interestingly, through the last decades, the role of lactate has changed drastically from being a byproduct of glucose—after anaerobic oxidation—to being considered as a neuroenergetic and signaling molecule [14,15]. However, no previous study has examined the antidepressant effects of lactate in a menopausal rat model.

In view of the above, since ovarian hormones regulate the neurotransmitter levels in the brain, it was suggested to use an ovariectomized rat model to eliminate the endogenous role of estrogen, followed by immobilization to induce stress in animals, to develop a depression-like model. Previous evidence has addressed the point that a low estrogen level alone is not enough to develop depression in menopausal women [2,16]. Therefore, this led us to perform immobilization in order to magnify the depression-like symptoms in our animal model.

In this study, we aimed to investigate the antidepressant-like effect of lactate and elucidate its mechanisms in ovariectomized and repeatedly stressed female rats. Depression-like behavior in rats was assessed by tail suspension test (TST) and forced swimming test (FST). Furthermore, its neuronal mechanisms were evaluated by utilizing an enzyme-linked immunosorbent assay (ELISA) of serotonin (5-HT) and dopamine (DA) and immunohistochemistry of tyrosine hydroxylase, (TH) tryptophan hydroxylase (TPH), and protein kinase C (PKC).

## 2. Experimental Section

### 2.1. Animal

Two experiments were conducted on 8 week old adult female Sprague–Dawley (SD) rats weighing 200–220g, which were obtained from the licensed breeder Samtako Animal Company (Seoul, Korea). Animals were housed with five rats per polycarbonate cage and were maintained under artificial light during a 12-h light/dark cycle (light 07:00–19:00) each day, with the temperature kept at 25 ± 2 °C and the humidity at 55 ± 15%; clean drinking water and a standard diet were provided ad libitum during the experiment.

All efforts were made to reduce the animals’ suffering. The study protocol has been strictly accomplished according to Guide for the Care and Use of Laboratory Animals from the National Institutes of Health (revised in 2011) and was approved by Kyung Hee Medical Center Institutional Animal Care and Use Committee (KHMC-IACUC), with approval number: KHUASP(SE)-18-073 (approval date 30 May 2017).

### 2.2. Experiments Schedule

#### 2.2.1. 1st Experiment

This study was designed to investigate the role of lactate on behavioral tests and its effect on neurotransmitter levels in different brain areas. Forty-two rats were randomly divided into the following 5 experimental groups: non-operated and non-stressed group (normal, *n* = 8), ovariectomized and stressed group (control, *n* = 10), ovariectomized and stressed with lactate 250 mg/kg treated group (lactate 250, *n* = 8), or with lactate 500 mg/kg treated group (lactate 500, *n* = 8), and ovariectomized with stress and treated with estradiol 1 µg/kg subcutaneously (E2, *n* = 8).

#### 2.2.2. 2nd Experiment

This experiment aimed to study the exact mechanism of lactate-induced upregulation in neurotransmitter levels. Thirty-four rats were randomly divided into 4 experimental groups: normal (*n* = 8), control (*n* = 10), lactate 100 mg/kg (*n* = 8), and lactate 250 mg/kg (*n* = 8).

**Figure 1:**
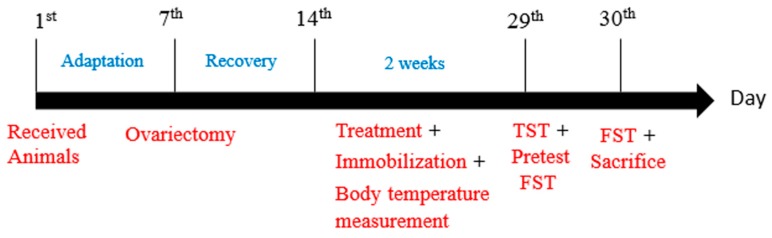
An experimental schedule for ovariectomy, oral lactate administration, and behavioral tests. Sprague–Dawley (SD) female rats were received and allowed to acclimatize themselves for one week. Consequently, all rats, except the normal group, were subjected to ovariectomy and allowed one week for post-surgery recovery. Therefore, from the 1^st^ day of treatment for two continuous weeks, lactate groups received their doses, and then, after 30 min, immobilization began. During the lactate receiving period, cutaneous body temperature was measured on the 2nd, 5th, 12th, and last day of treatment. All rats were subjected to a tail suspension test (TST) and forced swimming test (FST) on the 29th and 30th day, respectively. After the behavioral testing, the rats were sacrificed and brain tissues were immediately collected for analysis.

The second experiment was conducted after the finalization of the data analysis of the 1st experiment. Both experiments were performed under similar conditions (Figure 1); however, the rat brain extraction method was different according to the intended tests.

### 2.3. Drug Administration

From the 1st day of treatment, lactate groups were daily treated with lactate (l-(+)-lactic acid ≥98%, L1750, Sigma-Aldrich Chemical Co. St. Louise, MO, USA) with three different doses (100, 250, 500 mg/kg orally), normal and control groups were given sterile saline, and the estradiol group (β-estradiol ≥98%, Sigma-Aldrich Chemical Co.) received their treatment subcutaneously (1 µg/kg). All drugs were administered orally using a rat gavage needle and were freshly prepared just before every experiment for 2 continuous weeks. Immobilization then began 30 min after the treatments.

### 2.4. Ovariectomy Surgery (OVX)

Under sterile conditions and general anesthesia with pentobarbital sodium (50 mg/kg intraperitoneal), bilateral ovariectomy was performed. The surgical area was cleaned using ethanol 70%, and a midline dorsal skin incision of 2 cm was made using a surgical scalpel blade no. 11, according to the method described by Lasota et al. [17]. Using a surgical retractor, the ovaries were identified, then gently pulled outside the abdomen and removed with their fat using cautery.

After this, the uterine horn was returned to the abdomen; the incision was closed in two layers using sterile sutures. The muscle layer was stitched up using absorbable suture while the skin was closed by non-absorbable suture (Blue Nylon sutures-4/0, AILEE Comp. Ltd., Busan, Korea).

Lastly, the incision was wiped with povidone iodine for disinfection. All rats were housed individually for 1 day and then allowed 1 week for post-surgery recovery under laboratory conditions.

### 2.5. Immobilization Stress

After 7 days of postoperative recovery, the ovariectomized rats were stressed daily for 14 consecutive days according to chronic immobilization stress (CIS) protocols [18,19,20]. The stress was induced by forcing the rats into plastic bags (a disposable rodent restraint cone, Harvard Instrument, Holliston, MA, USA) for 2 h per day (10:00—12:00 P.M.) with no access to food or water. The cone-shaped plastic bags were designed to prevent any movement of the rat, while a large hole in the apex zone allowed the rat to breathe. All experimental groups, except normal, received the same restraint stress.

### 2.6. Tail Suspension Test (TST)

The TST used was carried out 24 h after the end of immobilization, as described in Can et al. [21]. Briefly, adhesive tape was folded several times around the proximal part of the rat’s tail. The free end of the adhesive tape (6 cm long) was attached to a metal hook which was positioned in the middle of the top of each compartment in a wooden box. The wooden box (height: 54 cm, width: 30 cm, and diameter: 47 cm) contained 4 compartments, which allowed testing 4 rats at one time. The width and depth of each compartment did not allow the rats to make contact with the walls and prevented rats from observing other animals while being tested. Rats were recorded for 6 min, and the immobile time was measured manually. Higher scores indicated depression in rodents.

### 2.7. Forced Swimming Test (FST)

This test took place in a transparent Plexiglas cylinder (20 cm diameter × 50 cm height) filled to a depth of 30 cm, with the water temperature at 25 °C. On the 15th day after treatment, the rats in all groups were adjusted for an initial 15 min pretest by placing them in water-filled cylinders. After 24 h, animals were subjected to 6 min of forced swim test. Immobile behavior was recorded with a video camera for later manual scoring. FST was based on a method described by Yankelevitch-Yahav et al. [22], with some modification. Immobility behavior is the time when an animal did not show any further attempts to escape. After the test, rats were dried and placed back in their cages. The water in the cylinders was changed between rats to maintain similar conditions.

### 2.8. Cutaneous Body Temperature Measurement

Peripheral skin temperature was measured on the 2nd, 5th, 12th, and last day of treatment. A small Rodent Infrared Thermometer (153-IRB, Braintree Scientific, Inc. Lab Research Product, Braintree, MA, USA) was used to measure the base of a rat’s tail skin temperature, which is similar to the method of this study with some modification [23].

### 2.9. Enzyme-Linked Immunosorbent Assay (ELISA) for Serotonin (5-HT) and Dopamine (DA) Measurements in Hypothalamus, Hippocampus, and Prefrontal Cortex

ELISA tests for serotonin and dopamine were performed on rats in the first experiment. After behavioral tests, rats anesthetized with pentobarbital sodium (80 mg/kg intraperitoneally) were decapitated. The hippocampus, prefrontal cortex, and hypothalamus were obtained rapidly from the rat brains and stored at −80 °C to measure 5-HT and DA levels.

The hippocampus, hypothalamus, and prefrontal cortex were homogenized in a lysis buffer (containing 137 mM NaCl, 20 mM Tris (pH 8.0), 1% NP40, 10% glycerol, 1 mM PMSF, 10 mg/mL aprotinin, 1 mg/ml leupeptin, and 0.5 mM sodium vanadate) by using a tissue homogenizer and then incubated at 4 °C for 1 min while shaking. Homogenates were centrifuged, and supernatants were collected. The total concentrations of 5-HT and DA in many brain areas were measured by an ELISA kit (Serotonin or Dopamine Research ELISA, catalog number: BA E-5900 and BA E-5300 respectively, Labor Diagnostika Nord (LDN), Nordhorn, Germany) according to the manufacturer’s protocol.

### 2.10. Immunohistochemistry for Tyrosine Hydroxylase (TH), Tryptophan Hydroxylase (TPH), and Protein Kinase C (PKC)

All immunohistochemistry analysis was conducted on the second experiment groups. After sacrificing the rats, the brains were removed, post-fixed over-night, and cryoprotected with 20% sucrose in phosphate-buffered saline (PBS) solution at 4 °C. By using a cryostat (Leica CM1850; Leica Microsystems Ltd., Nussloch, Germany), coronal sections (30 μm thick) were obtained from the hypothalamus and locus coeruleus (LC). The sections were immunostained for TH, TPH, and PKC expression by using the avidin-biotin-peroxidase complex (ABC) method.

Briefly, the sections were incubated with either primary mouse anti-TH antibody (Monoclonal antibody (1:1000) dilution; TH [24]: sc-136100, Santa Cruz Biotechnology, Dallas, TX, USA), or with primary sheep anti-TPH antibody (Polyclonal antibody (1:500) dilution; catalog number: AB1541, Merck KGaA, Darmstadt, German), or with primary mouse anti-PKC antibody (Monoclonal antibody (1:500) dilution; anti-PKC antibody (MC5) (ab31), Abcam, Cambridge, UK) in PBST solution (PBS plus 0.3 % Triton X-100) for 72 h at 4 °C. Next, the sections were incubated at room temperature for 2 h with secondary antibodies (1:200 dilution, Vector Laboratories Co.; Burlingame, CA, USA) in PBST containing 2% normal horse or rabbit serum, respectively.

The sections were then incubated for 120 min in ABC reagent (Vectastain Elite ABC kit, Vector Labs. Co., Burlingame, CA, USA), and then in a 3,3′-Diaminobenzidine (DAB) Enhanced Liquid Substrate System tetrahydrochloride (DAB; Sigma-Aldrich Chemical Co., Darmstadt, Germany) for 1 min. Finally, the tissues were washed in PBS solution and fixed individually onto slides. Images were captured using an Olympus BX53 microscope (Olympus Scientific Solutions Americas Corp., Waltham, MA, USA) and processed using CellSens Dimension software version 2.1 (cellSens Dimension, Inc.; Waltham, MA, USA).

The sections were viewed at 40×, 100×, and 200× magnification, and the expression of TH in the LC region and TPH and PKC in the hypothalamus were measured. The counted sections were randomly chosen from equal levels of consecutive sections. Moreover, the cells within the LC and hypothalamus areas were counted on each of the four sections per animal.

Counting of the immunopositive cells was performed within a square (150 × 150 µm^2^), localized in the LC (Bregma −9.72 mm, Interaural −0.72 mm) and hypothalamic regions (Bregma −2.40 mm, Interaural 6.60 mm) according to the stereotactic rat brain atlas of Paxinos and Watson (5th edition). Distinct brown or violet spots indicated TH, TPH, and PKC-immunoreactive cells in the LC and hypothalamus, respectively. The TH and PKC–immunopositive cells were counted by measuring their densities using imageJ1 software (Wayne Rasband, software for scientific image analysis, Bethesda, MD, USA); however, TPH–immunopositive cells were counted separately by a blind observer.

### 2.11. Statistical Analysis

All of the results from behavioral, biochemical, and immunological tests in both experiments are presented as mean ± standard error of the mean (SEM). Statistical analysis was performed using SPSS 23.0 software (SPSS Inc., Chicago, IL, USA). One-way analysis of variance (ANOVA) was followed by a post hoc Least Significant Difference (LSD) test in order to identify statistically significant changes in the biochemical and behavioral data. Normality of distribution and equality of variances were confirmed using these tests. *p* values ≤0.05 were considered statistically significant.

## 3. Results

### 3.1. Oral Lactate Administration Induced Antidepressant-Like Effect in TST

**Figure 2:**
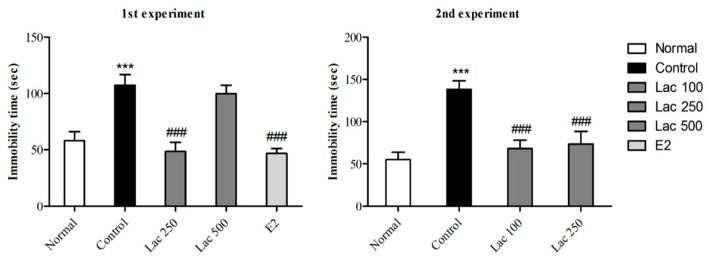
Effect of oral lactate administration on ovariectomized rats with stress in tail suspension tests (TST). Oral lactate administration (100 and 250) and estradiol decreased immobility time significantly compared to the control group. Values are presented as mean ± standard error of the mean (SEM). *** show the difference between the normal and control groups, while ### show significant differences between the control group and treatment groups. *** and ### *p* < 0.001. The *p* value of the test was < 0.001 by one-way ANOVA, followed by a post hoc LSD test.

After 14 days of continuously administered oral lactate, TSTs were performed in both experiments. The control group exhibited longer times spent immobile compared to normal group (Figure 2). However, lactate 100, 250, and estradiol administration significantly decreased immobility time in rats compared to control group (1st experiment *F*(4, 38) = 11.872, 2nd experiment *F*(3, 32) = 11.524, *p* < 0.001).

### 3.2. Oral Lactate Administration Decreased Immobility Time in FST

**Figure 3:**
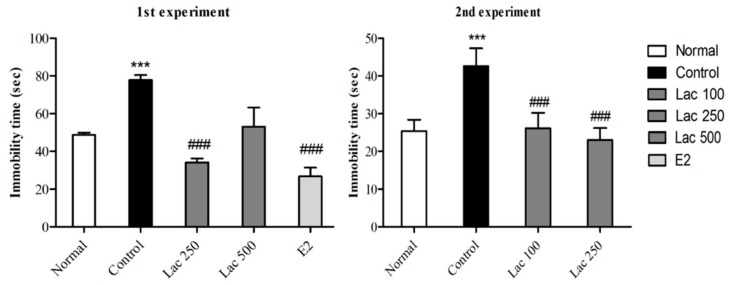
Oral lactate administration reduced immobility time in FST following ovariectomy and repeated stress. One-way ANOVA comparisons revealed a remarkable decrease in the time spent immobile in forced swimming tests in lactate treated groups (100 and 250) and the estradiol group compared to control rats. Values are presented as mean ± SEM. *** show differences between the normal and control groups. ### show significant differences between the control group and treatment groups. *** and ### *p* < 0.001. The *p*-value of the test was <0.001 by one-way ANOVA, followed by a post hoc LSD test.

In both experiments, ovariectomized and stressed rats exhibited longer times spent immobile. However, post hoc comparisons revealed a remarkable reduction in immobility of the lactate 100, 250, and estradiol groups compared to the control group (1st experiment *F*(4, 35) = 13.433, 2nd experiment *F*(3, 31) = 4.899, *p* < 0.001, Figure 3). The lactate 500 group did not show any significant reduction in immobility time in both TST and FST.

### 3.3. Oral Lactate Administration Produced a Reduction in Cutaneous Body Temperature in Rats with OVX and Stress

**Figure 4:**
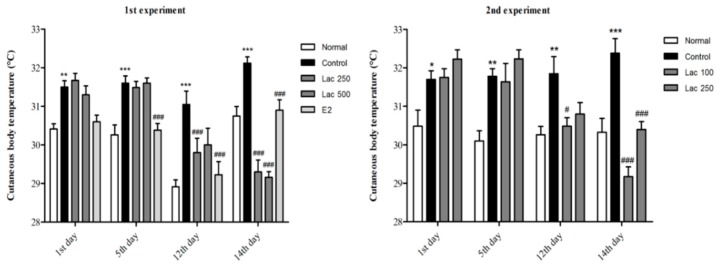
Oral lactate administration induced a reduction in cutaneous body temperature in rats with ovariectomy surgery (OVX) and stress. Control groups exhibited a gradual increase in cutaneous temperature; however, the estradiol and all lactate groups showed a notable decline in the cutaneous body temperature, especially in the last day of treatment. Values are presented as mean ± SEM. *, ** and *** show differences between the normal and control groups, while # and ### show significant differences between the control group and treatment groups. # *p* < 0.05 and ### *p* < 0.001. The *p*-value of the test was <0.001 by one-way ANOVA, followed by a post hoc LSD test.

In both experiments, the control group exhibited a gradual increase in cutaneous body temperature after ovariectomy and immobilization compared to the normal group (*p* < 0.001). All lactate groups and the estradiol group showed a significant decline in the cutaneous temperature, especially in the last day of the treatment receiving period (1st experiment *F*(4, 39) = 24.105, 2nd experiment *F*(3, 32) = 33.167, *p* < 0.001, Figure 4).

### 3.4. Oral Lactate Administration Elevated 5-HT and DA Concentrations in the Hypothalamus, Hippocampus, and Prefrontal Cortex (PFC)

**Figure 5:**
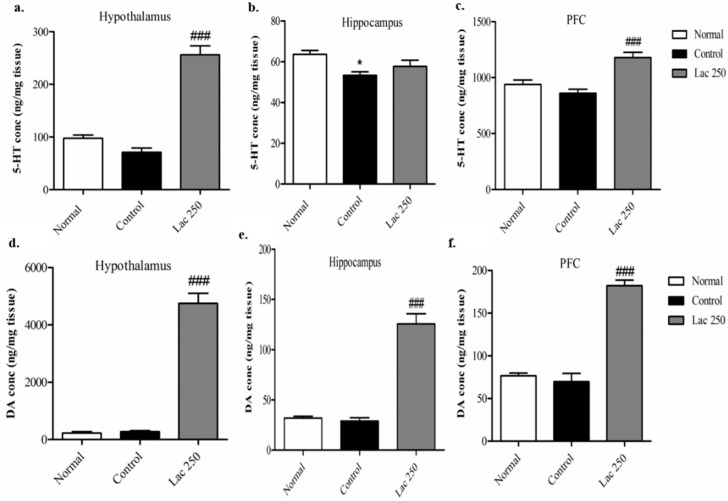
Effect of oral lactate administration on serotonin (5-HT) and dopamine (DA) concentrations in different brain areas in ovariectomized rats with stress. The levels of 5-HT concentration (nanogram per milligram) in the hypothalamus (**a**), hippocampus (**b**), and prefrontal cortex (**c**) changed after repeated lactate administration. The levels of DA concentration (nanogram per milligram) in the lactate 250 group in the hypothalamus (**d**), hippocampus (**e**), and prefrontal cortex (**f**) (*n* = 6). Values are presented as mean ± SEM. * show differences between the normal and control groups, while ### show significant differences between the control and treatment groups. ### *p* < 0.001. The *p*-value of the test was <0.001 by one-way ANOVA, followed by a post hoc LSD test.

ELISA analysis was conducted on rats’ brains from the 1st experiment, and since the lactate 500 group showed no significance in the behavioral tests, only the lactate 250 group underwent ELISA analysis. ELISA demonstrated that repeated lactate administration in the lactate 250 group significantly increased the total concentration of 5-HT in the hypothalamus and PFC compared with rats in the control group ((*n* = 6), *F*(2, 17) = 59.559, *F*(2, 16) = 12.890, *p* < 0.001, Figure 5a–c). Moreover, the lactate 250 group produced a remarkable upregulation in the total levels of DA in the hypothalamus, hippocampus, and PFC compared to the control group ((*n* = 6), *F*(2, 17) = 116.911, *F*(2, 18) = 59.140, *F*(2, 15) = 59.375, *p* < 0.001, Figure 5d–f).

### 3.5. Oral Lactate Administration Enhanced TH Expression in Locus Coeruleus (LC)

**Figure 6:**
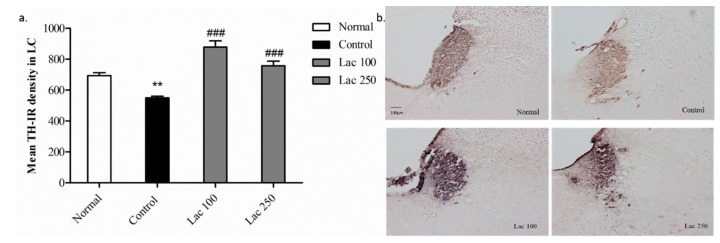
Tyrosine hydroxylase positive (TH+) cell expression in the locus coeruleus (LC) after oral lactate administration. (**a**) Oral lactate administration increased the mean TH- immunoreactive (TH-IR) cell density in ovariectomized rat with repeated stress in comparison to the control group. Values are presented as mean ± SEM. ** show differences between the normal and control groups. ### show significant differences between the control group and treatment group. ### *p* < 0.001. The *p*-value of the test was <0.001 by one-way ANOVA, followed by a post hoc LSD test (*n* = 6). (**b**) Representative images of TH+ cell expression in all groups in the LC region (magnification 100×). TH+ cell density was considerably increased in both lactate treated groups; however, it was denser in the lactate 100 group compared to the lactate 250 group.

In the 2nd experiment, we aimed to investigate the TH-immunoreactive (TH-IR) neurons in the LC area. In the control group, the mean TH-IR cells density was significantly downregulated compared with that in the normal group (*p* < 0.01). The decreased expression of TH-IR cells in the control group was considerably restored in the lactate 100 and 250 groups ((*n* = 6), *F*(3, 20) = 25.129, *p* < 0.001, Figure 6a). Similarly, this over-expression of TH positive cells was obvious with increased densities in the lactate groups’ tissues (Figure 6b).

### 3.6. Oral Lactate Administration Enhanced Immunohistochemistry Reaction of TPH in Hypothalamic Nuclei

**Figure 7:**
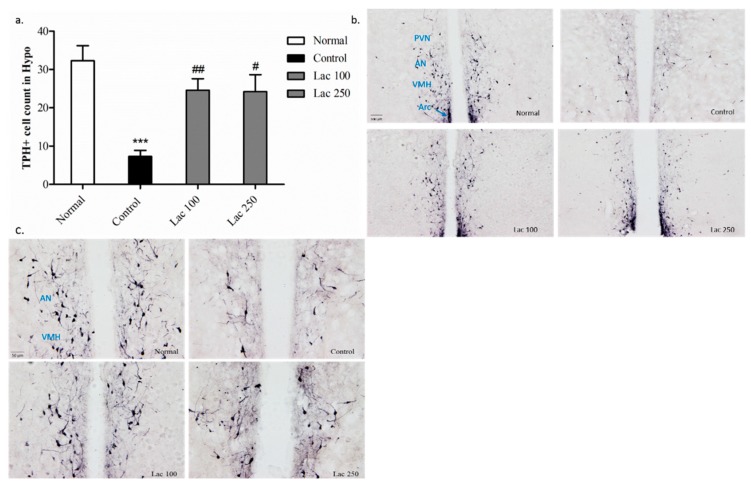
Oral lactate administration enhanced tryptophan hydroxylase (TPH) expression in the hypothalamic nuclei. (**a**) The decreased number of TPH+ cells in the control group was notably upregulated in the lactate 100 and 250 groups. (**b**,**c**) Representative images of TPH+ cell expression in the hypothalamic nuclei (the scale bars represent 100 and 50 μm, respectively). PVN = paraventricular hypothalamic nucleus, AN = anterior hypothalamic nucleus, VMH = ventromedial hypothalamic nucleus, Arc = arcuate nucleus. Values are presented as mean ± SEM. *** show differences between the normal and control groups, while # and ## show significant differences between the control group and treatment groups. # *p* < 0.05 and ## *p* < 0.01. The *p*-value of the test was <0.001 by one-way ANOVA, followed by a post hoc LSD test (*n* = 6).

In the control group, the number of TPH+ cells was diminished in the hypothalamus compared with that in the normal group (*p* < 0.001). The decreased number of TPH+ cells was significantly upregulated in the lactate 100 groups (*p* < 0.01) and 250 group (*p* < 0.05) ((*n* = 6), *F*(3, 21) = 10.688, Figure 7a). This over-expression of TPH+ cells was distinct in the lactate groups’ tissues in the paraventricular hypothalamic nucleus (PVN), anterior hypothalamic nucleus (AN), ventromedial hypothalamic nucleus (VMH), and arcuate nucleus (Arc) (Figure 7b,c).

### 3.7. Oral Lactate Administration Upregulated TPH Expression in Peduncular Part of the Lateral Hypothalamic (PLH)

**Figure 8:**
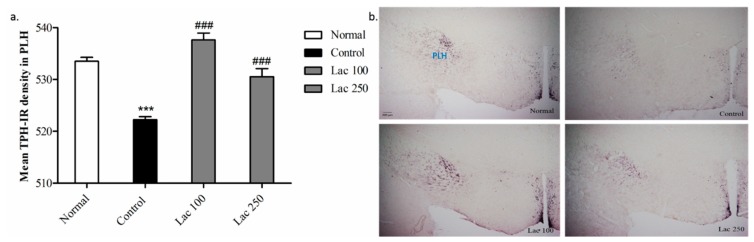
Changes occurred in TPH expression in the peduncular part of the lateral hypothalamic (PLH) following oral lactate administration. (**a**) The mean TPH-IR cell density was significantly increased in both the lactate 100 and 250 groups in comparison to the control group. Values are presented as mean ± SEM. *** show differences between the normal and control groups, ### show significant differences between the control group and treatment groups. ### *p* < 0.001. The *p*-value of the test was <0.001 by one-way ANOVA, followed by a post hoc LSD test (*n* = 6). (**b**) Representative images of TPH+ cell expression in lateral hypothalamic region (magnification 40×).

Expression of TPH antibodies in PLH was significantly decreased in the control group compared with that in normal group (*p* < 0.001). The attenuated expression of TPH in the control group was significantly enhanced in both the lactate 100 and 250 groups ((*n* = 6), *F*(3, 22) = 4.485, *p* < 0.001, Figure 8a). This over-expression of TPH+ cells was apparent with increased density in both lactate groups’ tissues (Figure 8b).

### 3.8. Oral Lactate Administration Regulated PKC Expression in Hypothalamic Area

**Figure 9:**
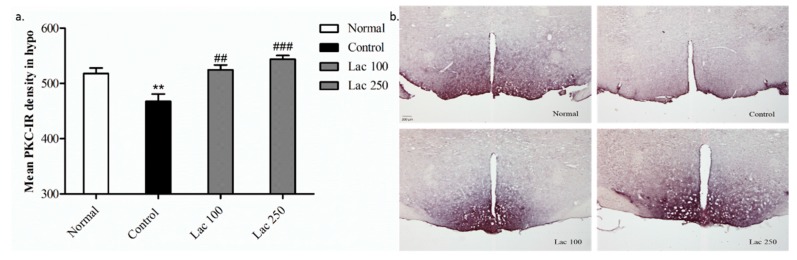
Oral lactate administration regulated protein kinase C (PKC) expression in the hypothalamic area. (**a**) Both the lactate 100 and 250 groups significantly upregulated the mean PKC-IR cell density in the hypothalamus (*n* = 6). (**b**) Representative images of all groups for PKC+ cell expression in hypothalamic region. PKC density was markedly increased in both lactate groups compared to the control group (the scale bar represents 200 µm). Values are presented as mean ± SEM. ** show differences between the normal and control groups, while ## and ### show significant differences between the control group and treatment groups. ## *p* < 0.01 and ### *p* < 0.001. The *p*-value of the test was <0.001 by one-way ANOVA, followed by a post hoc LSD test.

The expression of PKC-immunopositive cells in the control group showed diminished density in comparison to the normal group (*p* < 0.01). However, lactate administration significantly restored this decreased expression of PKC+ cells in the lactate 100 (*p* < 0.01) and 250 groups (*p* < 0.001) ((*n* = 6), *F*(3, 23) = 12.438, Figure 9a). Additionally, this over-expression of PKC-IR cells was noticeable by their increased density in both lactate groups’ tissues (Figure 9b).

## 4. Discussion

A recent study [25] provided evidence that peripheral administration of lactate (intraperitoneally) in the corticosterone-induced depression in mice model elevated hippocampal extracellular levels of lactate after 5 min of administration, measured by an inserted l-lactate selective biosensor in the hippocampus, and did not produce any significant effect on locomotor activity and neuromuscular strength. However, no previous study or literature reviews showed the effects of oral lactate administration in the modulation of monoaminergic neurotransmitters or in improving depression symptoms in the menopausal rat model.

In our study, behavioral tests were performed to evaluate the effects of lactate administration in rats. In TST and FST for both experiments, the control group showed a remarkable increase in time spent immobile compared to the normal group, which is consistent with the results of this study [16]. However, the lactate 100, 250, and estradiol groups decreased immobility time in the TST and FST, which is more likely a result of neurotransmitter modulation in different brain areas.

In the 1st experiment, the lactate 500 group showed no significant reduction in immobility time in both TST and FST, which is supported by the results of a study indicating that applying a high concentration of l-lactate via microdialysis leads to suppression of hippocampal neurons rather than stimulating them [26]. Therefore, the lactate 500 group was not investigated further in both the ELISA and immunohistochemistry tests.

A literature review reported that diminished estrogen hormones, along with abnormal NE levels, is known to cause thermoregulatory and vascular dysfunction, which lead to the occurrence of hot flushes. This is as a result of the influence of the noradrenergic system that affects the central sympathetic nervous system, which in turn affects the cutaneous and core blood flow [4,5,6,27]. Recent studies proved that surface body temperature measuring is a good alternative for core body temperature in animals. Peripheral body temperature measurement is inexpensive, noninvasive, and can serve as a reliable and sufficient alternative for implantable systems for measuring changes that occur in body temperature [23,28,29].

In our study, following ovariectomy and repeated stress, an infrared thermometer calculated a gradual increase in cutaneous body temperature in the control group, which indicates that these rats are experiencing hot flushes, consistent with the results of this study [30]. However, administration of lactate, as well as estradiol, showed a significant gradual reduction in cutaneous body temperature by 2∼3 °C, especially in the last day of treatment. Although it is difficult to clarify the exact mechanism, such a phenomenon seems to be a result of the changes observed in the enzymes associated with monoamine neurotransmitter synthesis [31,32].

Many studies have addressed the relationship between the levels of serotonin and dopamine and brain functions, including controlling mood and anxiety [33,34,35]. In order to investigate the changes observed in neurotransmitter levels after oral lactate administration, ELISA tests for serotonin and dopamine were conducted on the groups of the 1st experiment targeting three different brain areas (the hypothalamus, hippocampus, and PFC). Since the lactate 500 group did not show any significance in the behavioral tests, only the lactate 250 group underwent ELISA analysis. We found that the level of 5-HT was significantly increased in the hypothalamus and prefrontal cortex area. Additionally, the level of DA was drastically elevated in all of the three previously mentioned brain areas. It is worth mentioning that the levels of serotonin and dopamine between all normal and control groups were different; however, the huge elevation of the neurotransmitter levels in lactate groups made the difference between the normal and control groups not significant. This difference is only remarkable in hippocampal serotonin since lactate administration did not induce a huge elevation. In accordance with these studies [36,37], the authors reported that l-tryptophan and l-tyrosine (5-HT and DA precursors) administration to the stressed mice upregulated the levels of serotonin and dopamine remarkably in the hypothalamus and hippocampus areas. 

Therefore, in the 2nd experiment, we aimed to investigate the exact mechanisms of lactate that elevated the levels of dopamine and serotonin and induced antidepressant-like effects. For this purpose, an immunohistochemistry study was carried out by using anti-tyrosine hydroxylase (TH) antibodies in the locus coeruleus (LC) region. TH is the enzyme responsible for converting tyrosine to l-dopa, which is a precursor for the synthesis of dopamine, norepinephrine, as well as epinephrine. More evidence reported that noradrenergic locus coeruleus-tyrosine hydroxylase positive axons (LC-TH+) lead to DA release in the hippocampus, medial prefrontal, and parietal cortex. Also, either a reduction in LC-TH+ expression or norepinephrine transporter blockading in the LC region inhibits the release of DA, which signifies that LC terminal neurons can release both DA as well as NE [38,39,40,41,42,43]. Along with the results of this study [44], our data presented that the LC region is affected by stress (as in the control group) and exhibited a hypo-density of TH expression in the LC region. However, both the lactate 100 and 250 groups showed a significant increase in TH positive cell density in the LC region. This result could explain the increase happened in dopamine levels within many brain areas after lactate administration.

In addition, an anti-TPH antibodies assay was conducted in the hypothalamic region to elucidate the mechanism by which lactate-induced upregulation in serotonin levels occurs. Tryptophan hydroxylase is the neuro-enzyme involved in the biosynthesis of serotonin from tryptophan. Moreover, it is well known that serotonergic neurons in raphe nucleus project to innervate many hypothalamic nuclei [45]. Ovariectomized rats with no treatment (control group) presented with low TPH+ cell numbers in the hypothalamus, which is similar to the results of this study [24], whereas the lactate 100 and 250 groups restored these cells, especially in the paraventricular hypothalamic nucleus **(PVN), anterior hypothalamic nucleus (AN), ventromedial hypothalamic nucleus (VMH), and arcuate nucleus (Arc).

Moreover, TPH+ cell expression in the peduncular part of lateral hypothalamic nucleus (PLH) in the control group showed a lower density compared to the normal group. The low density in the lateral hypothalamic nucleus was drastically enhanced in both lactate treated groups. Overexpression of tryptophan hydroxylase in many hypothalamic nuclei after oral lactate administration indicates the mechanism of serotonin level upregulation.

All previously presented data show that the changes observed in TH and TPH activity can lead to subsequent alterations in the levels of serotonin and dopamine released in the brain [46]. The increases in TH and TPH expression were ultimately accompanied by an upregulation in the availability of serotonin and dopamine.

It has been recognized that decreased PKC activity is associated with patients affected by major depressive disorders [12,13,47]. Many reports have addressed the interaction between low TH and TPH levels and low PKC activity [48,49,50]. Therefore, in order to evaluate the role of repeated lactate administration on the modulation of PKC levels, we further performed immunohistochemistry analysis of anti-PKC antibodies in the hypothalamic region. PKC+ cell expression in the hypothalamic region in the control group displayed a lower density compared to the normal group. This low density of PKC immunoreactive cells in the hypothalamus was markedly upregulated in both the lactate 100 and 250 treated groups.

Collectively, all previously mentioned findings suggest that the antidepressant action of lactate was achieved by activation of PKC, leading to the upregulation of TH and TPH expression and, eventually, enhancement of serotonin and dopamine levels in the brains of menopausal rats. All of these results were measured by behavioral, immunological, and biochemical tests. Future researchers should further investigate the interaction between lactate and neuronal cells.
